# Editorial

**Published:** 1858-12

**Authors:** 


### Editorial.

### EXTRACTING TEETH—ELECTRO-MAGNETISM.

This subject still continues to attract much attention. The main point does not yet seem to be very definitely settled, some embracing one opinion, some another in regard to it. There is but little known, really in regard to it as yet, though many are experimenting with it, and doubtless in a very little while much more will be known about it. Of the manner in which it prevents pain, in the extraction of teeth, or any surgical operation, we have heard two or three theories, rather hesitatingly suggested; nothing however, that bears with it much evidence of its correctness.

Some have supposed it to be the nervous excitement, diverting the attention from the operation. They suppose they find proof of this in the fact, that in some cases in which it seems to be successful at first, after a time it loses its effect. All who have experimented with it, have found some cases, in which it did not produce the desired results. This is sometimes the case, in the first operation, and in subsequent operations the desired result is produced. Upon examination failures will be found to depend upon one of three things. The patient may be an unfavorable one constitutionally.

1st. There are some constitutions, to which even the feeblest galvanic current is intolerable, even more so than the extraction of a tooth. We have found two or three cases, in which the passage of a slight current through a tooth, would cause the patient to scream with pain, in such we could not expect to succeed in removing a tooth without pain. In other cases we have applied it, and the patient represented it,—even a considerable current—as rather a pleasant sensation. One gentleman remarked, "put it on stronger, there is not enough to feel good," and there was a pretty strong current on at the time; then we expected a success and we *had it.* The different susceptibilities of different constitutions, to the influence of electro magnetism, is perfectly familiar to every one who has experimented with it.

2nd. The tooth may be in an unfavorable condition, one expresses the belief that periostitis is unfavorable for the exhibition of electro-magnetism.

In almost every case where there is diseased periosteum there is pain, and whether in such it mitigates it at all is a question. I have in one case removed a large molar tooth, thus affected, under the influence of galvanism without pain, and in one case removed a superior lateral incisor without pain, when there had been intense pain, from inflammation of the periostium for fifteen hours.

3rd. Intense pain may be produced in almost any case, by an awkward application. In any case the current should be tested upon the patient, before the application is made to the tooth, and no stronger current should be given, than is pleasant to the patient; that will usually be a correct criterion; the hands of the patient should be moist when making this test, otherwise it might not be a true one. Every case of failure, I have traced to one of these three causes.

It is but seldom that the constitutional idiosyncrasy above referred to is met with. The second difficulty of course may occur more or less frequently, dependent upon circumstances, beyond the control of the operator.

Another theory, one more ingenious if not more true, is founded upon the fact, that the nerves are the channels of communication between the brain or source of sensation, and other parts, and it is then assumed that electricity is the agent of communication, as upon the telegraphic wire; that the nerves are indeed so many telegraphic wires upon which the subtile agent plays, and that when a stronger current, though it be an artificial one, is thrown on to them, it counteracts the weaker, though it be the natural one, and that the circle of the stronger current, being completed outside of the brain, the weaker current is neutralized, intelligence, of even great injury, would not then be communicated to the seat of sensation. This theory may be squinting in the direction of a solution, but as it is presented now, it is liable to some objections. If this theory was literally correct, we would only have to include any part of the system in an electro-magnetic circuit, to produce insensibility to any operation. I have no theory to present upon this subject. Electricity in the various forms, in which it is presented, is not as tangible as some other things; it is one of the most, if not the most subtile material in nature. We understand but little of its *maneuvers* upon an iron wire, and when it comes to play upon the vital parts, and upon instruments of so peculiar a character as the nerves, about which we know so little, we hope we will be pardoned for not presuming to know all about it. *Nerves, electricity* and *vitality*, all thrown together, and then to account for the modus operandi of the manifestations, is rather more than we have attained to as yet. In connection with this, I will give two or three cases, which will present about the ordinary results, according to our observations.

*Case* 1.—Nov. 11—Mr. J. presents the first left superior molar for extraction. He is of a nervous sanguine temperament, very irritable and fearful of pain. The tooth had been aching from an exposed nerve, and very severely. He remarked that his teeth were very hard to draw, and he wished to have the battery applied, if there was any hope or probability of relief. The battery was applied and the tooth removed, and his first remark was, "Why sir, that is perfectly astonishing, it did not give me one particle of pain." He left the office perfectly delighted with the operation. The tooth was firmly set and required a strong effort to remove it.

*Case* 2d.—Mrs S., delicate; aged 26, of a nervous constitution, presented three molar teeth for extraction. She desired the application of the galvanic current. The gum was separated about one, when she became very sick and faint. Cold water was dashed upon the face, and camphor applied to the nostrils as restoratives, without much effect; applied galvanism through the arms, slight at first, increasing it gently, till the current was as strong as could pleasantly be borne. She was at once completely restored, and seemed to be much stimulated by the current, and had three large teeth removed, with far less pain than she had ever experienced in a similar operation. She remarked that after this, the extraction of a tooth would have no terror for her if she could have galvanism. She felt more vigorous after the operation than before it.

*Case* 3rd.—Mr. McC., of bilious temperament, in good health; removed the second superior left bi-cuspid, pulp dead, no periostitis, had never ached. It was removed without any pain whatever, with battery attached. Has had other teeth extracted but they gave pain. These cases present about the ordinary results in the extraction of teeth under the influence of electro-magnetism, in none of these was there much pain. I have on record two or three cases in which there was considerable pain, but they were dependent upon circumstances to which reference is already made.

It is important in the application that the forcep should not touch the tongue or lips. The most simple method of preventing this, is with a piece of pure rubber tube three fourths of an inch in diameter and three inches long, slipped on the joint of the forceps. We know nothing better than this. We would not be without the battery and would desire all to try it.

We are frequently asked about the right to use it; in regard to this, all have about the same facilities for obtaining information, and we know no more about it than others may.

All we have to say is, do not infringe upon the well defined rights of any, but always be found upon the side of progress and improvement. T.

---

### THE AMERICAN JOURNAL ON DENTAL CHEMISTRY.

*The American Journal* is again in trouble on the chemistry of caries. It says in reference to the discussions on this subject at Cincinnati, "The unfounded theories about nitric acid in the mouth played a conspicuous part;" and again, "it was said that nitric acid produced upon teeth out of the body, appearances resembling white decay."

No doubt the above was said, for the American Journal says so, but we are utterly at a loss to know who said it. It was probably said only in a whisper; for we were present at all the discussions, and listened with a good degree of interest. Indeed, about the only member of the convention that said anything on the subject was *ourself*, known as the "anonymous friend" of the Journal. And if the above quotation has any allusion to our remarks, we have only to say that we did not speak of the action of nitric acid *out of the body;* for our experiments were tried *in the mouth* as well as out of it. And we did not then, and do not now claim "that nitric acid is the sole agent in the production of the white decay;" but we did claim that nitric acid is capable of producing white decay either in or out of the mouth, and we stated then that as yet we "knew of nothing else capable of producing it." Will the American Journal have the kindness to tell us of something else capable of producing it?

The Journal tells us "there are, indeed, very good reasons for doubting the presence of free nitric acid in the mouth. The hypothesis of the conversion of ammonia into nitric acid will not answer at all. The conditions under which this change takes place do not exist in the mouth."

Well, doctors differ. It maintains on the authority of Bischoff, that "nitric acid in a free state, is formed in nature, only when an electric spark passes through oxygen and nitrogen."

Liebig tells us that the last products of the decay and putrefaction of animal bodies are *ammonia*, in the temperate and cold climates, and *nitric acid* in the tropics and hot climates, and he adds that the latter is the product of the transformation of the former. The mouth is not a *tropic*, but the Journal will agree thats its *climate* is warm.

Liebig also tells us that, "when moist azotized animal matter is exposed to the action of the air, ammonia is liberated;" and again he says "ammonia can not be exposed to the action of oxygen without the formation of an oxyd of nitrogen, and in consequence the production of nitric acid." This latter idea is variously stated by him, as for example, "nitric acid is always a product when ammonia is present in the substance exposed to oxydation."

From this it is evident that Liebig believes that the hypothesis of the conversion of ammonia into nitric acid *will answer*, notwithstanding the contrary opinion of the Journal.

In speaking of this transformation of ammonia, the Journal says, "It is no easy matter, to accomplish this result;" but Liebig says, "It is owing to the *great facility* with which ammonia is converted into nitric acid, that it is so difficult to obtain a correct determination of the quantity of nitrogen in a compound subjected to analysis, in which it is either contained in the form of ammonia, or from which ammonia is formed by an elevation of temperature."

The Journal says of this transformation that "the surrounding deoxydizing influences render it almost impossible that it could occur" in the mouth.

The *deoxydizing* powers of the mouth may sound strange to those dentists who use oxydizable metals either for plates or fillings; but then they must yield the point that these powers are quite formidable, for the Journal is not likely mistaken, though it did once tell us that none but cane sugar is used in food. But in what do these deoxydizing influences consist? Are they explained by the fact that the saliva absorbs oxygen readily from the air and *imparts it to other bodies*? that, on this principle, it oxydates gold and silver, when triturated with it? (See Piggot's Chemistry p. 172.) Now, every dental student knows that substances are oxydized more readily in the mouth than in the atmosphere; and we conclude, there fore, that the junior editor must have indited this, for the senior, being a practical dentist, would know better than to make such a statement.

It is pretty evident the Journal and we differ in regard to the chemistry of caries. We have only to say that on our part, and we presume on theirs too, there is an honest difference. And though we have not referred to many authorities in these remarks, it is not that they are scarce, but that brevity is desirable. The positions of Liebig above noticed are sustained by chemists generally—not by all we are well aware. And if these positions are true, we contend that we have given the most rational account of "white decay" on record. Every dentist knows that decay is usually a gradual process; and every chemist knows that the constant formation of nitric acid for a length of time, in quantities too small to be recognized by chemical tests, would be sufficient to produce the disorganization of the tooth. And if the same state of circumstances on a large scale invariably produces nitric acid, we have a right to infer that these circumstances, on a reduced scale, will produce the same results, correspondingly reduced.

In conclusion we do hope the Journal will tell us what does produce dental decay in its various forms, or at least attempt to give us some rational account of the matter. One of the editors has told us that "the different acids generated during the putrefaction or fermentation of vegetable or animal food are fully capable of disorganizing the teeth." Is this true of carbonic acid? one of the acids generated. (See Piggot's Chemistry, p. 157.) Now we submit that it is time we had something of an affirmative nature from the Journal. It has been negative long enough. It is easy for it to deny the soundness of our positions. Will it give us its own? W.

---

### THE AMERICAN JOURNAL AND THE AMERICAN DENTAL CONVENTION.

In regard to the late meeting of the American Dental Convention, the *American Journal* says: "For the summary of the proceedings of the Dental Convention, held at Cincinnati in August last, we are indebted to a professional friend who was present on the occasion;" and further it says, "Not having a copy of the papers read on the occasion, we are unable to publish any of them in the present number of the Journal. We may copy some of them from other Journals in our next issue."

We are sorry the papers were not received by the Journal; and that our readers may know that the fault is not ours—that we have not acted selfishly in the matter—we would state that, in accordance with a previous agreement, we sent advance sheets of all the papers read at the Cincinnati meeting, the *official* minutes, and *our own* report of the discussions. These were forwarded to the American Journal more than two months' before its arrival in Cincinnati; and the Register was forwarded about a week later.

But there is something funny in the history and character of the Journal's report of the proceedings of the late meeting. Now, that "professional friend," to whom it is indebted for its "summary of the proceedings" took but few, if any notes, as all who were at the Convention could testify if we would FRANK-ly tell them who he is, (and we could tell.) What a fine memory he has! especially for the long speeches. And it is pretty good even on the short ones, except that it has led him in most instances to transpose the ideas. The reader will cease to be surprised that the "professional friend's" report is only a reproduction of ours, sadly mutilated, when we tell him that that same "professional friend" wrote to us soon after his return home, for advance sheets of the proceedings, and that they were promptly forwarded to him. He was undoubtedly pleased with our report of his own remarks as they are almost invariably transferred *erbatim et literatim.*

We will only add that the Journal might have had *our report* more accurate by obtaining it more directly. It would have been heartily welcome to it;—it is welcome to it even in the mutilated form in which it has reproduced it. But what are we to expect from a Journal which, after unjustly charging us with making an anonymous attack on its senior editor, has not the *manliness* to retract, after its mistake is pointed out to it? W.

---

### ETHER QUESTION.

DR. W. Y. G. MORTON has recently entered suit against Dr. Davis of the U. S. Marine Hospital at Chelsea, for infringing his patent for the use of Ether, as an anasthetic, damages laid $5,000. We supposed that this claim had been long ago abandoned. There are several circumstances that led us to this conclusion. Two men, (Drs. Morton and Jackson,) jointly swore out a patent as discoverers of it, neither of them having any just claim as discoverers of the anæsthetic properties of ether, for it seems Dr. Wills discovered it two years before the patentees. Again application was made to Congress by one of the patentees for remuneration, which he need not have done had he not virtually abandoned the patent as untenable: fearful that he might be unsuccessful in this, the aid and sympathy of friends in New York were sought, to procure him a subscription of ten thousand dollars, as a slight acknowledgment, of the "inestimable boon," conferred by him on mankind. It is affirmed that he personally made application to a member of the medical profession, for permission to enter an amicable suit against him, that he might with more hope of success prosecute his designs in regard to this matter, both in Congress and elsewhere.

From all these we could not avoid the conclusion that the patent was abandoned, but failing in every other effort to obtain emolument, this suit is instituted as a last resort; in this, as in his other efforts, we doubt not he will fail, as justly he should. But in the mean time he may harass and annoy a party that never did any injustice to Dr. Morton. But Dr. Davis will have the sympathy and aid too, if he needs it, of every right minded member of the medical profession. T.

---

The Dental Reporter is still *reporting progress.* Toland is still alive and is likely to *report* himself quarterly. The Reporter gives a true index of the state of dental manufactures, as its proprietor is always up with the times in this department. The Reporter is publshed at 25 cents per annum. Those who can not afford to subscribe, should take the Lamp, or wait for the issue of some cheap journal. W.

*The Cincinnati Dental Lamp*, edited and published quarterly, by J. M. Brown, of the Cincinnati Dental depot. Price, 20 cents a year in advance,

The Lamp is neatly executed and contains, including advertisements, thirty-six pages. Its original department is mainly indebted to the industry and talents of the editor. His remarks are varied and spicy, characteristic of Dr. J. M. Brown. Its subscription price is within the reach of all. Who starts the next one? W.

*The Cant Hook.*—A Quarterly Journal, devoted to the extraction of teeth, the rolling of logs, and *the advancement of the Proprietor's business.* Published by E. Saw-Miller, Editor and Proprietor. "Not received *in exchange.*"

This Journal contains two pages (or rather is contained between two,) neatly executed and well filled with original and selected matter. A writer of mature age and great experience is engaged to contribute to its original department, and should his energies fail, hosts of younger authors are ready to take his place. The "*Cant Hook*" is published at the exceedingly low price of ten cents per annum, "*invariably in advance*,' unless the subscriber should prefer to receive it as the proprietor's advertising medium, and pay the postage on it wholly for "the advancement of our glorious profession."

*Prospectus—The Cent-er Drill.*—The Cent-er Drill will be a sizable Journal, issued semi-occasionally or oftener, and as its name imports, will be *blunt* at one end and *sharp* at the other. It will be used in boring holes through the incrustations which incase professional fogyisms, that "a light to dispel the darkness" may be enabled to illuminate their interiors. Nothing will be admitted to our pages unless it is sharp pointed, or at least *good on a bore.*

Terms.—To be within the reach of all, and *to defy competition*, the Drill will be issued at the low price of one cent per annum, and will be cheerfully loaned to "decided bores," and all other *hole*-souled fellows.

Address, X. K. VATOR, Proprietor.

*The Quarterly Journal of Dental Science for October*,—Published by Walton & Maberly, London, is at hand, with more than it usual interesting and instructive variety. On one of our advertising pages, will be found a synopsis of its contents, which, however, does not give much of an idea of the real character of the articles which it contains. The profession in England, just at this time, occupies rather an interesting attitude, and one which will have much influence upon the profession in the United States, as well as in England. Every intelligent dentist will feel that he should keep posted in regard to these things· This can only be done by taking one of the English Journals. Their proceedings are too voluminous for transfer to our pages. Every dentist will find the Quarterly Journal a valuable acquisition to his periodical literature. It can be had of Lindsay & Blackiston, Philadelphia.

☞ The following persons have consented to act as agents for the Dental Register. They will distribute the numbers to the subscribers in their respective places; receive subscriptions and receipt for the same, also take new subscriptions and transact any business pertaining to it.

E. A. L. Roberts, 55 Bond Street, New York,
Bingham, care of Jones, White & McCurdy, Chicago, Ill.,
Wm. T. Copeland, care of Fisk & Hall, Cleveland, O.,
Dr. A. M. Leslie, St. Louis, Mo.,
Dr. D. D. Smith, Syracuse, N. Y.,
Dr. A. Blakesley, Utica, N. Y.,
Dr. J. M. Brown, Cincinnati, O.,
J. T. Toland, Cincinnati, O.,
S. F. Dawes, Louisville, Ky.

We call attention to the following notices and would urge all invited to attend.

INDIANAPOLIS, October, 1858.

*Dear Sir:*—We, the undersigned, practicing Dentists of the City of Indianapolis, on consultation at a preliminary meeting held at the office of Dr. Jno. F. Johnston, have determined to call a meeting of the Profession, at Indianapolis, on Tuesday, December 28, 1858, for the purpose of forming a State Dental Association. We have done this, believing that we would be placed on more friendly and pleasant terms with each other; and that, by the discussion of such subjects, giving our experience in the treatment of unusual cases, the presentation for examination of peculiar casts, instruments, &c., &c., that it would be mutually advantageous; and, also, because we have been urged to do so by several of the profession from other portions of the State, whose names you find below, whom we had the pleasure of meeting at the American Convention. We, therefore, earnestly request you to co-operate with us, and bring whatever models, casts, reports of cases, &c., &c., that you may have, that will be of interest to the meeting. A number of prominent members of the Profession, from other States, have promised to be with us; and believing that nearly all in our own State will be here, we anticipate a pleasant and instructive holiday meeting. We feel we must not be behind our neighbors in this respect; we therefore trust you will come. Should you know of any of your professional acquaintances not receiving this Circular, will you do us the favor to invite them.

| | |
|---|---|
| G. C. NORTH, Indianapolis, | J. P. ULREY, Rising Sun, |
| T. M. NICHOLS, " | W. R. WEBSTER, Richmond. |
| JOHN MOFFITT, " | Dr. LUPTON, Shelbyville. |
| O. H. KENDRICK, " | Dr. GOODE, Madison, |
| JNO. F. JOHNSON, " | Dr. MARTIN, Franklin, |

Contributors to the Register, Vol. 12—The following gentlemen have very kindly volunteered as contributors for the Register:

Dr. James Taylor; Dr. H. R. Smith; Dr. W. B. Roberts; Dr. A. Berry; Dr. C. B. Chapman; Dr. Jos. Richardson; Dr. Wm. C. Kallum; Dr. D. Pratt; Dr. W. W. Allport; Dr. E. Collins; Dr. I. B. Branch; Dr. C. C. Dills; Dr. W. H. Shadoan; Dr. C. Bonsall; Dr. H. A. Smith; Dr. George Watt; Dr. J. Taft; Dr. S. F. Compton; Dr. Wise; Dr. Fisher; Dr. J. Murray; Dr. J. McCalla.

---

### HARRIS—NEW EDITION.

The Principles and Practice of Dental Surgery, By Chapin A. Harris, M. D., D. D. S., Seventh Edition, Enlarged and Improved, has just come to hand, from the popular publishing house of Lindsay & Blackiston, Philadelphia. This work we find really enlarged, containing 892 pages, and has been improved, we may say, much improved. We do not intend to say that the former edition was imperfect,—it was all that could be expected at that time. The improvements which we notice in this edition, are based upon the improvements and progress made in dental practice, since the issue of the last edition. These seem to have been very fully and appropriately incorporated into the work.

We think that the work is now quite large enough, for a text work of this kind; and when it is issued again, we hope it will be at least in two volumes. No dentist, who wishes to have the standard authorities upon dental practice, can afford to be without the latest edition of this work. It should have a place in the library of every physician.

The work is for sale by Rickey, Mallory & Webb, No. 143 Main Street. Price, $4. Also all the standard dental and medical works will be found upon their shelves.

---

### COMPLIMENTARY SUBSCRIBERS.

Reader, did you ever hear of them? We know you never did. They may have been in existence a long time, but they were not known to science till we lately discovered them, while engaged in earnest research for something far more profitable.

We discovered one of them in this way, and all are discovered by nearly the same process.

A dentist, in the full tide of successful practice, had received the *Dental Register*, regularly, for years; and when his bill was presented, he stated that he thought the *Register* had been sent to him merely for the sake of having prominent dentists on the subscription list. He never supposed he was expected to pay for it,—supposed it was desirable to have his influence, etc.

Now, this man took the *Register* on the published terms, and his bill was more than once forwarded to him; yet he attempted to *defraud* the publisher, in the way we have described. As we can't think of any term stronger than *unmitigated meanness*, we will not attempt to christen his conduct. On behalf of the publisher, we can say, that if any one should receive the *Register* as a compliment, it will not be the dentist in full practice, and, therefore, well able to pay for it,—but the worthy *beginner* who is struggling with the *truths and trials* of his profession. The only way a man can be an honor to a subscription list, is to *pay in advance*.

W.

Dr. Elisha Townsend, of Philadelphia.—The death of this worthy gentleman will give pain to a large circle of friends; and by his death science has lost a most intelligent servant. He was President of the Dental College, Philadelphia, and has been instrumental in doing much to advance the true interests of the profession throughout the country. As a man, he possessed noble qualities; and as a friend, he was prompt, unselfish and sincere. He had recently visited Europe for his health, but returned only to die. He will long be remembered by those who knew him, and the absence of his aid and counsel will make a void not easily filled. *Boston*, Oct. 18th, 1858. E. G. T.

---

At a meeting of the Faculty of the Pennsylvania College of Dental Surgery, held October 19th, 1858, the following resolutions, presented by Dr. McQuillen, were unanimously adopted:

*Resolved*, That we have heard, with profound emotion, of the decease of our late eminent and distinguished colleague, Prof. Elisha Townsend, whose uniform courtesy and affability endeared him to us as an associate, whilst his abilities as a practitioner and teacher, commanded our unqualified respect.

*Resolved*, That, throughout a long career, during which he enjoyed a most enviable position in the estimation of the profession and the community, and cherished a constant and deep interest in the advancement of the former, he contributed largely by the soundness of his judgment, the high order of his practice, and the elevated tone of his sentiments, in raising its standard to the position occupied at present.

*Resolved*, That the active and prominent part he maintained, and the sacrifices he made for years, to advance the interests of the Dental profession, justly entitle his memory to the grateful consideration of every practitioner.

*Resolved*, That we render to his widow, in her bereavement, our deep, heartfelt sympathies.

*Resolved*, That a copy of these resolutions be communicated to the widow, and the Dental Magazines be furnished with a copy for publication. T. L. Buckingham, Dean.

By the above, it will be seen that the dental profession has lost one of its brightest ornaments,—one who has ever been foremost in every good work, for the elevation of the profession to which he was so ardently attached. He stood high above any thing like professional jealousy; he was always desirous to take by the hand any professional brother, and lift him up, if need be, to as nearly his own level as possible. His motto was "*level up*," not level down, and he always acted upon this principle. He has filled many places of trust and honor in his profession, and always with the most brilliant success. His integrity, both as a man and as a member of his profession, was of the highest order. If, in any instance, he found himself in a wrong position, he with true magnanimity abandoned it, and warned others of the danger; in no case have we seen him maintain a false position, simply because he was in it.

His loss will be sensibly felt wherever he is known, but especially in the dental profession throughout the world.

